# Ocular Surgery in Patients With Severe Atherosclerotic Disease: Anesthetic Considerations and Vascular Risk Stratification

**DOI:** 10.7759/cureus.106968

**Published:** 2026-04-13

**Authors:** Saul J Prado Fonseca, Fabiana M Araya Padilla, Nicole Rodríguez, Natalia Ruiz Rojas, Jose Ignacio Chacon Soto, Gabriel Anchía Jiménez

**Affiliations:** 1 Emergency Medicine, Hospital Monseñor Sanabria Martínez, Puntarenas, CRI; 2 General Medicine, Caja Costarricense de Seguro Social (CCSS), Alajuela, CRI; 3 General Medicine, Hospital Monseñor Sanabria Martínez, Puntarenas, CRI; 4 General Medicine, Universidad de Costa Rica, Alajuela, CRI

**Keywords:** anesthesia, atherosclerosis, ocular perfusion, ocular surgery, perioperative management, vascular risk stratification

## Abstract

Ocular surgery in patients with severe atherosclerotic disease represents a growing clinical challenge due to the increasing prevalence of cardiovascular comorbidities in aging populations. Atherosclerosis, characterized by endothelial dysfunction, inflammation, and arterial stiffness, compromises vascular autoregulation and reduces ocular perfusion, thereby increasing susceptibility to ischemic complications during perioperative hemodynamic fluctuations. In this context, patients undergoing ophthalmic procedures, even those considered low risk, such as cataract surgery, may experience significant cardiovascular and cerebrovascular events.

A comprehensive preoperative evaluation is essential and should include detailed cardiovascular history, functional capacity assessment, frailty evaluation, and the use of validated risk indices. Targeted investigations such as electrocardiography, echocardiography, and carotid Doppler imaging allow for the identification of high-risk features and guide perioperative planning. The management of antithrombotic therapy requires an individualized approach that balances thrombotic and bleeding risks, particularly in patients receiving dual antiplatelet therapy or anticoagulation.

The choice of anesthetic technique plays a critical role in maintaining hemodynamic stability. Techniques with minimal systemic impact, such as topical anesthesia and Sub-Tenon block, are generally preferred, while general anesthesia should be reserved for selected cases. Intraoperatively, maintaining stable mean arterial pressure, avoiding hypotension and hypertension, and ensuring normocapnia are key strategies to preserve cerebral and ocular perfusion.

Postoperative care should focus on early detection of myocardial ischemia, arrhythmias, and neurological complications, as well as timely resumption of antithrombotic therapy. Ultimately, a multidisciplinary and individualized approach is essential to optimize perioperative safety and improve outcomes in this high-risk population.

## Introduction and background

The aging population is experiencing a progressive increase in atherosclerotic disease, with cardiovascular disease representing the leading cause of death in adults over 75 years of age. In this context, atherosclerosis is understood as a progressive and systemic condition that affects multiple vascular territories, including the coronary and carotid arteries, thereby significantly increasing the risk of ischemic events [1].

Severe atherosclerotic disease is defined by the presence of conditions such as multivessel coronary artery disease, carotid stenosis greater than 70%, and a prior history of stroke or transient ischemic attack [1]. These clinical scenarios have direct implications for surgical outcomes, as they are associated with a higher incidence of perioperative complications, particularly stroke and myocardial infarction [2].

Although ocular surgeries are primarily localized procedures, they may have relevant systemic implications in patients with severe atherosclerosis. Interventions such as cataract surgery, glaucoma surgery, and vitreoretinal procedures require careful evaluation of the patient’s cardiovascular status to minimize perioperative risk and optimize outcomes [1]. In addition, ocular tissues are particularly vulnerable to ischemia because adequate ocular perfusion depends on the balance between systemic blood pressure and intraocular pressure, while structures such as the retina and optic nerve are especially sensitive to reductions in blood flow [3].

In this population, the perioperative period is characterized by an increased susceptibility to adverse events, including stroke, myocardial infarction, and mortality [2]. This risk is further illustrated by evidence showing that octogenarians undergoing carotid endarterectomy present a slightly higher incidence of perioperative stroke compared to younger individuals, underscoring the importance of thorough risk stratification [2]. Moreover, the presence of comorbidities such as coronary artery disease amplifies the likelihood of major adverse cardiovascular events during and after surgical procedures [3].

Given these considerations, appropriate perioperative management becomes essential. Preoperative evaluation should include a detailed assessment of cardiovascular status, with particular attention to carotid stenosis and coronary artery disease [3]. During surgery, strategies should focus on maintaining adequate cerebral perfusion and stable blood pressure to reduce the risk of ischemic complications [4]. Postoperative care should prioritize close monitoring for early signs of stroke, myocardial infarction, and other complications, allowing for timely and appropriate interventions [5].

The objective of this review is to provide a comprehensive analysis of anesthetic considerations and vascular risk stratification in patients with severe atherosclerotic disease undergoing ocular surgery. It integrates pathophysiological foundations, preoperative evaluation, intraoperative management strategies, and postoperative care to optimize perioperative safety, reducing the incidence of cardiovascular and cerebrovascular events, and improving clinical outcomes in this high-risk population.

## Review

Methodology

This paper was developed as a structured narrative review aimed at providing an updated and clinically integrated analysis of anesthetic considerations and vascular risk stratification in patients with severe atherosclerotic disease undergoing ocular surgery. The review was conducted in accordance with the Scale for the Assessment of Narrative Review Articles (SANRA) framework and followed a predefined methodological approach established before literature screening [6]. It is important to note that this study was not designed as a systematic review and, therefore, does not adhere to PRISMA (Preferred Reporting Items for Systematic Reviews and Meta-Analyses) guidelines. Given the clinical heterogeneity of this patient population, the variability in cardiovascular risk profiles, and differences in anesthetic techniques and ophthalmic procedures, a narrative interpretative synthesis was selected over quantitative pooling [6]. This approach allowed for the integration of vascular, anesthetic, and ophthalmologic perspectives into a coherent and clinically applicable framework, with emphasis on perioperative cardiovascular risk, hemodynamic management, and clinical outcomes.

A comprehensive literature search was performed using PubMed, Scopus, and Web of Science, including peer-reviewed articles published in English or Spanish between January 2020 and December 2025. The final search was conducted in March 2026. This timeframe was selected to capture contemporary advances in perioperative risk stratification, anesthetic management, and cardiovascular optimization in ophthalmic surgery. Foundational studies were incorporated when necessary to contextualize key pathophysiological mechanisms and established clinical practices. The search strategy combined Medical Subject Headings and free-text terms using Boolean operators related to ocular surgery, anesthesia, atherosclerosis, carotid artery disease, coronary artery disease, perioperative risk, vascular risk stratification, antithrombotic therapy, hemodynamic management, and perioperative complications. Searches were conducted across titles, abstracts, and indexed subject headings to maximize sensitivity.

**Figure 1:**
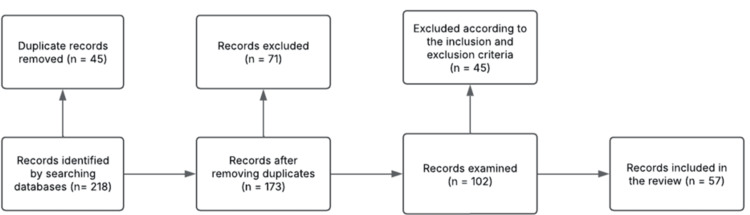
Flow diagram of literature search and study selection. Image credit: All authors (Lucid chart)

The initial search yielded 218 records. After removal of duplicates, 173 articles underwent title and abstract screening. Of these, 102 studies were selected for full-text evaluation, and 57 were included in the final qualitative synthesis. Study selection was performed independently by two authors, with disagreements resolved through discussion and consensus. Given the narrative nature of the review, study selection was guided by predefined inclusion and exclusion criteria rather than a systematic screening protocol (Figure 1).

To enhance transparency in the selection process, a flow diagram was incorporated to illustrate the identification, screening, eligibility, and inclusion phases. Additionally, predefined eligibility criteria were established before screening and are summarized in Table 1.

**Table 1:** Eligibility criteria for study selection.

Category	Inclusion criteria	Exclusion criteria
Population	Adult patients with atherosclerotic disease undergoing ocular surgery	Pediatric populations or patients without atherosclerotic disease
Clinical focus	Studies addressing anesthetic considerations, perioperative management, or vascular risk stratification	Purely technical ophthalmologic studies without systemic or perioperative relevance
Outcomes	Cardiovascular, cerebrovascular, hemodynamic, or ocular complications	Studies without clinical or perioperative outcome data
Study design	Randomized controlled trials, observational studies, systematic reviews, meta-analyses, guidelines, expert consensus	Case reports, editorials, letters, non–peer-reviewed publications
Language	Articles published in English or Spanish	Articles in other languages
Timeframe	January 2020-December 2025	Studies outside the timeframe (except foundational studies for the pathophysiological context)
Relevance	Direct evaluation of anesthesia, vascular risk, or perioperative outcomes in ocular surgery	Studies not addressing anesthetic or vascular considerations in the target population.

Eligible studies included randomized controlled trials, large observational cohorts, systematic reviews, meta-analyses, expert consensus statements, and contemporary international guidelines from anesthesiology, ophthalmology, and cardiovascular societies. Priority was assigned to multicenter studies, investigations with clearly defined cardiovascular risk stratification, and research evaluating perioperative outcomes such as stroke, myocardial infarction, hemodynamic instability, and mortality.

Data extraction focused on variables relevant to the clinical objectives of the review, including study design, patient comorbidities, cardiovascular risk profile, type of ocular procedure, anesthetic technique, perioperative management strategies, antithrombotic therapy, and reported outcomes such as stroke, myocardial infarction, hemodynamic instability, and mortality.

Methodological quality and risk of bias were assessed narratively according to study design and methodological characteristics. Randomized controlled trials and meta-analyses were considered to provide higher levels of evidence, whereas observational studies were evaluated with particular attention to potential selection bias, confounding variables, and heterogeneity in patient populations and interventions. Narrative reviews and expert consensus statements were recognized as having a higher inherent risk of bias due to the absence of standardized methodology. Across the included literature, the main sources of bias identified were clinical heterogeneity, variability in anesthetic techniques, and differences in outcome reporting. In cases of conflicting evidence, greater interpretative weight was assigned to higher-level evidence and guideline-supported recommendations.

Reference lists of included studies were manually screened to identify additional relevant publications. Given its narrative design, this review is subject to potential selection bias and does not provide pooled quantitative estimates. Artificial intelligence-based tools were used exclusively to assist in literature organization and structural coherence, while critical appraisal, synthesis, and final interpretation were conducted independently by the authors to preserve methodological rigor.

Surgical context and risk profiles in ophthalmology

Endothelial dysfunction represents an early and central event in the development of atherosclerosis and is characterized by increased vascular permeability and a pro-inflammatory state that promotes plaque formation and instability [7]. Within this pathological context, inflammatory mediators and macrophage infiltration contribute to plaque destabilization, thereby increasing the risk of atherothrombosis and intraplaque hemorrhage [8]. Because endothelial cells play a fundamental role in maintaining vascular homeostasis, their dysfunction also contributes to thromboembolic complications that may compromise ocular perfusion [7, 9].

As atherosclerosis progresses, arterial stiffness increases and impairs the autoregulatory capacity of blood vessels, including those supplying the eye [10]. This loss of autoregulation promotes fluctuations in ocular perfusion pressure and increases susceptibility to ischemic injury in the retina and optic nerve, particularly during periods of hemodynamic instability [11]. In addition, carotid artery stenosis, a frequent consequence of advanced atherosclerosis, can significantly reduce blood flow through the ophthalmic artery and further compromise ocular perfusion [12]. Hemodynamic simulations have shown that reductions in blood velocity and mass flow within the ophthalmic artery are associated with systemic atherosclerotic conditions such as diabetes mellitus and coronary artery disease, reinforcing the relationship between systemic vascular pathology and ocular circulation [13].

Ocular perfusion pressure, defined as the difference between arterial blood pressure and intraocular pressure, plays a fundamental role in maintaining adequate blood supply to ocular tissues [14]. In the setting of atherosclerosis, alterations in systemic blood pressure and increased vascular resistance may disrupt this balance and predispose patients to ocular ischemic events [11]. Retinal microvascular dysfunction, which reflects underlying endothelial impairment, has also been identified as a predictor of adverse cardiovascular events and may serve as an indicator of heightened vulnerability to ischemic damage during ocular surgery [15]. Intraoperative hemodynamic fluctuations may further aggravate ischemic stress in the retina and optic nerve, especially in patients with already compromised ocular perfusion [11].

Preoperative risk stratification and optimization

A thorough cardiovascular history is a fundamental component of preoperative evaluation, with particular attention to prior myocardial infarction, stroke, and previous revascularization procedures, as these factors are critical for estimating the risk of perioperative cardiac events [16,17]. Patients with a history of acute coronary syndrome or peripheral artery disease are often classified as very high risk for future cardiovascular events and therefore require more intensive evaluation and management [18].

Assessment of functional capacity also plays a central role in perioperative risk stratification. Functional capacity, commonly quantified in metabolic equivalents, has been consistently associated with postoperative outcomes, with lower values correlating with increased mortality and morbidity [19]. Frailty assessment adds further prognostic value, particularly in older adults, where instruments such as the Edmonton Frail Scale allow for a more refined characterization of physiological reserve and vulnerability [20].

Risk estimation can be strengthened through the use of validated indices. The Revised Cardiac Risk Index is widely used to estimate perioperative cardiac risk, and higher scores are directly associated with increased postoperative mortality [21]. In addition, the Surgical Apgar Score may improve predictive accuracy when used alongside the Revised Cardiac Risk Index, providing a more comprehensive evaluation of perioperative risk [22].

Targeted diagnostic investigations are essential to complement clinical assessment. Electrocardiography is routinely used to identify ischemic changes, while echocardiography provides detailed information regarding ventricular function and valvular disease [23]. In patients with suspected or established cerebrovascular disease, carotid Doppler ultrasound is particularly useful for assessing the degree of stenosis and plaque characteristics, both of which are major determinants of stroke risk [24].

The identification of unstable cardiac conditions is another critical step in preoperative evaluation. Recent acute coronary syndrome and decompensated heart failure significantly increase perioperative risk and may require postponement of surgery or further cardiology assessment [25]. Accordingly, preoperative optimization should focus on stabilizing modifiable risk factors, including blood pressure control, glycemic optimization, and correction of volume imbalances, all of which may reduce the likelihood of perioperative complications [24]. When unstable cardiovascular conditions persist, or further assessment is required to achieve adequate clinical status, surgery should be deferred until appropriate optimization has been completed [17, 25].

Antithrombotic and chronic medication management

Perioperative management of antithrombotic therapy requires careful balancing of thrombotic and bleeding risks. In the case of aspirin, continuation is generally favored in low-bleeding-risk procedures because of its protective effect against thrombotic events. Evidence from other surgical settings, such as coronary artery bypass grafting, suggests that maintaining aspirin therapy does not significantly increase bleeding risk, which supports a similar approach in ocular procedures with low hemorrhagic risk [26]. Nevertheless, this decision should not be generalized and must be individualized according to the patient’s thrombotic risk profile and the specific characteristics of the planned procedure [27,28].

Management becomes more complex in patients receiving dual antiplatelet therapy, usually consisting of aspirin plus a P2Y12 inhibitor, especially in those with coronary stents. The timing of interruption is critical because premature discontinuation is associated with a substantial risk of stent thrombosis. In this context, guided dual antiplatelet strategies based on platelet function testing or genetic profiling have been proposed to optimize the balance between bleeding and thrombotic risks. Some evidence suggests that withholding dual antiplatelet therapy for more than two days before surgery may reduce bleeding without significantly increasing thrombotic complications, although the certainty of this evidence remains limited [27,29].

Management of oral anticoagulants, including warfarin and direct oral anticoagulants, likewise depends on an individualized assessment of procedural bleeding risk and baseline thrombotic risk. In low-bleeding-risk procedures, continuation of anticoagulation may be acceptable, whereas in higher-risk interventions, temporary interruption with appropriate timing of discontinuation and resumption is required [30]. When anticoagulation is interrupted, bridging therapy with heparin may be considered in patients at high thromboembolic risk; however, this strategy must be weighed carefully because it is associated with increased bleeding [30].

In addition to antithrombotic agents, chronic cardiovascular medications require careful perioperative management. Beta-blockers should generally be continued to avoid withdrawal effects and associated cardiovascular complications. Statins are also typically maintained because of their pleiotropic vascular benefits beyond lipid lowering [30]. By contrast, angiotensin-converting enzyme inhibitors and angiotensin receptor blockers require individualized consideration, since they may affect intraoperative hemodynamic stability. Their continuation or temporary suspension should be determined on the basis of the patient’s overall clinical status and the expected hemodynamic demands of surgery [30].

Selection of anesthetic technique

Topical anesthesia is characterized by minimal systemic impact, making it an attractive option in patients with severe atherosclerotic disease. It is widely used in procedures such as cataract surgery and has been associated with a low complication rate of approximately 0.3% [31]. However, its success depends heavily on patient cooperation, which may limit its usefulness in anxious individuals or in those unable to remain still during the procedure [32].

The Sub-Tenon block has emerged as a favorable alternative because it carries a lower risk of vascular and neural injury compared with sharp-needle techniques such as peribulbar and retrobulbar blocks [33]. It also provides effective analgesia, with evidence demonstrating lower intraoperative and postoperative pain scores than peribulbar anesthesia [34]. In contrast, peribulbar and retrobulbar blocks are associated with a higher risk of hemorrhagic complications, particularly in patients receiving anticoagulation therapy, which makes them less suitable in severe atherosclerotic disease [31]. Retrobulbar anesthesia has also been shown to influence intraocular pressure and ocular pulse amplitude, potentially affecting ocular perfusion in patients with pre-existing retinal or optic nerve compromise [35].

General anesthesia remains indicated in uncooperative patients and in complex surgical procedures where local or regional techniques are insufficient [32]. However, its use entails systemic risks, including respiratory depression and possible alterations in cerebral perfusion, which require careful intraoperative management [36]. Sedation strategies must therefore be tailored to avoid respiratory depression and hypercapnia, as both may adversely affect cerebral hemodynamics and compromise perfusion [37]. Overall, anesthetic techniques associated with minimal systemic effects, such as topical anesthesia and Sub-Tenon block, are generally preferred in patients with severe atherosclerotic disease because they promote greater hemodynamic stability and may reduce the risk of ischemic complications [35].

Intraoperative hemodynamic and respiratory management

Maintaining stable mean arterial pressure is a central goal during intraoperative management, particularly in patients with severe atherosclerotic disease and impaired autoregulatory capacity. A mean arterial pressure of at least 60 mmHg is generally recommended to support adequate cerebral perfusion under these conditions [38,39]. Continuous arterial pressure monitoring is preferable to intermittent measurements because it enables more precise detection and management of both hypotensive and hypertensive episodes.

Avoidance of hypotension is especially important, since reductions in arterial pressure may significantly decrease cerebral blood flow and oxygen delivery, thereby increasing the risk of ischemic injury, including watershed infarctions [40]. Predictive tools such as the Hypotension Prediction Index have therefore been proposed to anticipate and prevent intraoperative hypotension, helping reduce both its incidence and duration [41]. At the same time, prevention of acute hypertension is equally important, as elevated blood pressure may increase the risk of plaque rupture in patients with atherosclerotic disease and thereby precipitate severe cardiovascular or cerebrovascular complications [39]. For this reason, blood pressure should be maintained within the limits of cerebral autoregulation to avoid excessive mechanical stress on vulnerable plaques [42].

Appropriate monitoring strategies are essential to achieve these hemodynamic goals. Standard noninvasive monitoring may be sufficient in lower-risk patients, but it may fail to detect rapid blood pressure fluctuations. In contrast, invasive arterial monitoring is recommended in high-risk individuals because it provides continuous and accurate measurements that facilitate timely therapeutic intervention [38].

Ventilatory management also plays a key role in preserving cerebral perfusion. Maintenance of normocapnia is essential, since deviations in carbon dioxide levels can induce cerebral vasoconstriction or vasodilation and thereby alter cerebral blood flow [43]. Fluid therapy must likewise be balanced carefully to avoid both hypovolemia and fluid overload, as either may destabilize hemodynamics and increase perioperative risk. Vasoactive agents are often necessary in this setting. Phenylephrine and ephedrine are commonly used to correct hypotension, and phenylephrine, as a selective α1-adrenergic receptor agonist, has been shown to increase cerebral microvascular perfusion and support cerebral oxygenation during hypotensive episodes [44]. The choice of vasoactive therapy should be individualized according to the patient’s hemodynamic profile and the underlying mechanism of instability [38].

Postoperative care and complication prevention

Patients with severe atherosclerotic disease are at increased risk of postoperative myocardial ischemia and arrhythmias, making early detection a critical component of postoperative care. Continuous cardiac monitoring is recommended to identify early signs of ischemia or rhythm disturbances and to allow timely intervention [45]. Preoperative cardiovascular evaluation also contributes to postoperative planning by guiding the intensity and duration of surveillance strategies [46].

Neurological assessment is equally important because of the elevated risk of perioperative stroke in this population. Early postoperative evaluation for signs of stroke or transient ischemic attack allows prompt diagnosis and treatment, which are essential for improving outcomes [47,48]. Preventive intraoperative strategies, including maintenance of optimal blood pressure and goal-directed therapy, may further reduce the incidence of neurological complications.

Ocular complications such as retinal artery occlusion and ischemic optic neuropathy must also be considered, since they are associated with an increased risk of subsequent cardiovascular and cerebrovascular events [28,49]. Protective intraoperative measures, including avoidance of excessive ocular compression, are important in minimizing these adverse outcomes [36].

Adequate control of pain and agitation is another essential aspect of postoperative care because insufficient control may lead to hemodynamic surges that exacerbate underlying cardiovascular disease. Multimodal analgesia and carefully titrated sedation protocols help reduce physiological stress and preserve hemodynamic stability [47]. Timely resumption of antithrombotic therapy is also important for the prevention of thromboembolic complications, although reintroduction must be balanced against the risk of postoperative bleeding. Notably, despite the increased incidence of stroke and myocardial infarction in patients with retinal artery occlusion, antithrombotic therapy has not demonstrated a clear protective effect during the first year, highlighting the need for individualized therapeutic strategies [50].

In the ambulatory setting, discharge criteria should include stable vital signs, adequate pain control, and the absence of neurological or cardiovascular complications. Patient education regarding warning signs and the importance of follow-up is essential to ensure safe recovery. Given the generally low incidence of severe complications in ophthalmic surgery, most patients can be discharged safely when appropriate monitoring and follow-up plans are in place [51].

Special clinical scenarios

Patients with high-grade carotid stenosis greater than 70% represent a particularly high-risk subgroup in the perioperative setting. In these individuals, carotid endarterectomy is generally preferred over carotid artery stenting because it is associated with a lower risk of stroke and restenosis, although carotid artery stenting may offer advantages such as a lower incidence of myocardial infarction and cranial nerve injury [52]. In patients undergoing coronary artery bypass grafting, the addition of carotid endarterectomy has not shown a significant effect on long-term outcomes compared with coronary artery bypass grafting alone, although it may increase perioperative risk [53].

In patients with a recent history of stroke or transient ischemic attack within the preceding three to six months, the timing of carotid intervention is a major determinant of outcomes. Early intervention, particularly within 48 hours, may be beneficial in patients with stable neurological status, whereas delays beyond 30 days have been associated with increased mortality [54]. Similarly, carotid artery stenting performed within the first 7 days or after 28 days following stroke has been linked to higher complication rates, underscoring the importance of careful timing [55].

Management of patients with severe coronary artery disease and limited functional reserve also requires individualized decision-making. In this group, combined strategies such as coronary artery bypass grafting with carotid endarterectomy have not consistently demonstrated superior outcomes compared with coronary artery bypass grafting alone and may be associated with increased perioperative risk [53]. Therefore, the choice between carotid artery stenting and carotid endarterectomy should be based on patient-specific risk profiles, with carotid artery stenting potentially favored in patients at higher risk of myocardial infarction [52].

Patients with concomitant atherosclerosis and heart failure represent an additional layer of complexity and require a multidisciplinary approach to optimize perioperative management. In such cases, impaired dynamic cerebral autoregulation may increase susceptibility to ischemic events; however, some evidence suggests that autoregulatory function may improve after carotid endarterectomy, indicating a possible benefit in stabilizing cerebral perfusion [56]. In patients undergoing combined ocular and vascular procedures, the risk of perioperative stroke may be reduced through careful preoperative optimization, including consideration of carotid revascularization in those with significant stenosis. Because of the increased complexity and higher potential for adverse outcomes, these combined interventions require meticulous planning and coordination to maximize patient safety [47].

## Conclusions

Ocular surgery in patients with severe atherosclerotic disease carries a significant risk of cardiovascular, cerebrovascular, and ocular complications due to the interaction between endothelial dysfunction, impaired vascular autoregulation, and decreased ocular perfusion. Therefore, rigorous preoperative risk stratification, along with optimization of risk factors and appropriate selection of anesthetic techniques, is essential to reduce adverse events and improve clinical outcomes.

Perioperative management in this population must be comprehensive and multidisciplinary, focusing on intraoperative hemodynamic control, continuous monitoring, balancing thrombotic and hemorrhagic risk, and early detection of postoperative complications. These strategies minimize the risk of cerebral and ocular ischemia and optimize safety in complex clinical settings.
